# Predicting Potential Treatment Targets for Fatigue in Chronic Fatigue Syndrome Using Thalamic Seeding

**DOI:** 10.1002/brb3.71428

**Published:** 2026-04-23

**Authors:** Kang Wu, Tian Zhou, Zhuoran Li, Sitong Feng, Ziyao Wu, Yanzhe Ning, Kuangshi Li, Hongxiao Jia

**Affiliations:** ^1^ Beijing Key Laboratory of Intelligent Drug Research and Development for Mental Disorders, National Clinical Research Center for Mental Disorders, National Center for Mental Disorders, Beijing Anding Hospital Capital Medical University Beijing China; ^2^ Advanced Innovation Center for Human Brain Protection Capital Medical University Beijing China; ^3^ Stead Family Department of Pediatrics University of Iowa Carver College of Medicine, Iowa City Iowa USA; ^4^ Dongzhimen Hospital Beijing University of Chinese Medicine Beijing China

**Keywords:** chronic fatigue syndrome, FC, fMRI, non‐invasive brain stimulation, occipital cortex, thalamus

## Abstract

**Background:**

Chronic fatigue syndrome (CFS) is a neurological disorder. Functional connectivity (FC) abnormalities have been implicated in fatigue symptoms, but candidate cortical targets for non‐invasive brain stimulation (NIBS) remain unclear.

**Methods:**

We recruited 100 CFS patients and 100 healthy controls (HC) and performed bilateral thalamic seeding to generate FC maps. Two approaches were used to identify candidate stimulation targets: normative analysis based on a public dataset of 1000 healthy individuals and individual analysis based on subject‐specific FC patterns. FC peaks were evaluated at both vertex and sphere levels and examined in relation to fatigue severity measured by the 14‐item Fatigue Scale (FS14). Finite element analysis (FEA) was used to estimate cortical regions potentially influenced by NIBS. In an additional longitudinal dataset of 35 CFS patients undergoing one month of Tai Chi exercise, we examined associations between exercise‐induced FC changes within these regions and changes in fatigue severity.

**Results:**

Normative analysis identified a negative FC peak in the left lateral occipital cortex (MNI: –51.0, –77.5, 7.0), near the PO7 electrode, whose FC values were associated with both fatigue severity and exercise‐related changes in fatigue. In contrast, individual analysis revealed substantial spatial variability in peak locations with low overlap across participants and inconsistent associations with fatigue severity.

**Conclusions:**

These findings suggest that thalamic–surface FC alterations are associated with fatigue symptoms in CFS and the normative analysis may provide a practical reference framework for identifying candidate cortical targets for future NIBS studies.

## Graphical abstract

Bilateral thalamic seeding identified a candidate cortical target near PO7 in the left lateral occipital cortex. Its FC correlated with fatigue severity and exercise‐induced fatigue in CFS, suggesting an FC‐guided framework for future NIBS targeting fatigue symptoms.

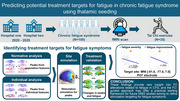

## 1 Introduction

Chronic fatigue syndrome (CFS) is a disorder characterized by long‐term, unexplained fatigue lasting more than six months, often accompanied by sleep disturbances, cognitive impairment, and post‐exertional malaise (PEM), ultimately leading to profound impacts on work, social activity, and daily life (Fukuda et al. 1994). The underlying mechanisms of CFS remain unclear, and standardized diagnostic criteria and effective treatments are still under investigation. Fatigue represents an integrated manifestation of abnormal physiological states, encompassing both physical and mental dimensions. One perspective views fatigue as a consequence of excessive energy consumption or impaired energy production, suggesting that CFS could be a metabolic dysfunction disorder. Mitochondria are the primary source of cellular energy and play a central role in sustaining body functions. Previous evidence has revealed mitochondrial abnormalities in CFS, including increased oxidative stress and disrupted redox balance (Mantle et al. 2024; Syed et al. 2025). Furthermore, supplementation with coenzyme Q10, a key factor in maintaining mitochondrial function, has been shown to be effective in alleviating fatigue symptoms in randomized clinical trials (Cordero et al. 2013; Mantle et al. 2024). Alternatively, CFS can also be considered a neurological disorder (Murga Gandasegui et al. 2021). Clinically, patients often present with cognitive impairments such as poor memory, difficulty concentrating, and reduced attention span (Bansal et al. 2025). From this perspective, fatigue is understood as a negative symptom of impaired brain function, potentially driven by immune dysregulation and central nervous system inflammation. Studies have revealed abnormal immune exhaustion in CFS patients, including dysfunction of T cells and natural killer (NK) cells (Maya 2023). Supporting this view, treatment with rituximab, an immune‐modulating agent, has shown beneficial effects in some patients (Sotzny et al. 2018). Overall, the multifaceted pathogenesis of CFS is gradually being recognized, and continued efforts are required to bridge the gap between mechanistic insights and clinical applications.

Advanced non‐invasive neuroimaging techniques have provided novel insights into brain dysfunction in CFS and begun to clarify the neural mechanisms underlying mental fatigue. Task‐based functional magnetic resonance imaging (fMRI) studies show that, during attention‐focused tasks, CFS patients recruit broader brain regions to achieve response times and accuracy levels comparable to healthy controls (Shan et al. 2018), with abnormal activity observed particularly in the brainstem (Washington et al. 2020) and cerebellum (Inderyas et al. 2023). In contrast to body energy generated by mitochondria, the concept of brain energy has emerged from advances in neuroscience. The brain operates as a large‐scale network, with cognitive processes such as decision‐making, working memory, and language processing relying on coordinated activity across multiple subregions (Ji et al. 2019). Under normal conditions, this network maintains an optimal architecture that enables rapid information transfer and efficient regional cooperation with minimal communication cost (Liao et al. 2017; Park and Friston 2013). When network hub integrity is disrupted, this architecture deteriorates, communication burden increases, and brain energy consumption rises to compensate (Wu et al. 2022). Functional connectivity (FC), which quantifies the relationships between brain regions, serves as a key measure of network communication efficiency. Resting‐state fMRI studies have revealed FC impairments in CFS patients, including disruptions within the brainstem (Barnden et al. 2019) as well as in the parahippocampal and occipital lobes (Gay et al. 2016), with these alterations correlating with fatigue severity. Our previous work further demonstrated that FC abnormalities in the frontoparietal network and default mode network, both of which are central to cognitive control and self‐referential processing, distinguished CFS patients from healthy controls with an accuracy of 80.5% (Wu et al. 2022). Accumulating evidence of FC disruption (Wortinger et al. 2017) and network breakdown (Kim et al. 2015) in CFS supports the brain energy theory and has drawn increasing attention to the role of FC in CFS pathophysiology and diagnosis. This naturally raises the question of whether defective FC can be used to guide treatment targets in CFS.

Non‐invasive brain stimulation (NIBS) techniques, such as transcranial magnetic stimulation (TMS) and transcranial direct current stimulation (tDCS), can induce electrical currents that modulate brain tissue and local neuronal excitability. By targeting a specific region, ideally the true focus of the disrupted network, NIBS can restore functional relationships between brain regions and optimize network architecture (Siddiqi et al. 2022; van Rooij et al. 2024). CFS is characterized by impaired neuronal excitability (Davey et al. 2003; Samii et al. 1996; Starr et al. 2000), and several clinical trials have shown that patients can benefit from NIBS through modulation of cortical excitability, improving symptoms such as fibromyalgia (Mhalla et al. 2010) and declined cognition (Deodato et al. 2024; Tiwari et al. 2024), although current evidence remains insufficient to strongly support the broad clinical application of NIBS for CFS. Two factors may contribute to the slow growth in this field. First, most previous studies applied TMS to alleviate fibromyalgia, largely overlooking fatigue as the primary symptom of CFS. In contrast, a study using gentle tDCS demonstrated significant improvements in subjective fatigue and vigilance decrement (Hanken et al. 2016), suggesting that tDCS may be particularly suitable for CFS patients with prominent fatigue. Second and more importantly, the effectiveness of NIBS critically depends on the stimulation site. Prior TMS studies targeted the motor cortex (Deodato et al. 2024; Mhalla et al. 2010) or dorsolateral prefrontal cortex (DLPFC) (Tiwari et al. 2024), while the tDCS study targeted the parietal cortex (Hanken et al. 2016). Consequently, identifying stimulation sites specifically targeting fatigue in CFS is crucial, not only to guide TMS and tDCS interventions but also to inform other NIBS approaches as alternative treatment strategies for CFS. Given that our previous work demonstrated a key role of disrupted FC in CFS, this study uses FC as a marker to identify optimal treatment targets, with the hope of advancing the brain energy theory of mental fatigue.

The thalamus plays a central role in the generation of mental fatigue. Studies have shown that thalamic dysfunction contributes to fatigue in multiple sclerosis (Capone et al. 2020), ischemic stroke (Wang et al. 2022), post‐COVID (Leitner et al. 2024), CFS (Barnden et al. 2019), and other disorders (Li et al. 2019). One potential pathological pathway involves the hypothalamic‐pituitary‐adrenal (HPA) axis, which regulates the transmission of signals from the peripheral nervous system to the central nervous system. Dysregulation of this axis can result in an abnormally heightened perception of effort and reduced endurance for sustained physical and mental activities (Chaudhuri and Behan 2004). In addition, studies using fecal metabolomics (Lupo et al. 2021) and magnetic resonance spectroscopy (MRS) (Thapaliya et al. 2025) have both demonstrated significantly elevated glutamate levels in CFS, and a randomized clinical trial further showed that a low‐glutamate diet alleviated fatigue symptoms (Holton et al. 2020). A previous hypothesis proposed that elevated extracellular glutamate may disrupt neuronal‐glial signaling in the central nervous system, leading to (1) increased excitability and higher cellular energy demands, and (2) reduced glutamate transmission that fails to meet these heightened demands, ultimately resulting in mental fatigue (Hansson and Rönnbäck 2004). Notably, glutamate is the principal neurotransmitter of thalamic neurons (Bevan et al. 1995). Taken together, this growing body of evidence strongly supports the rationale for using thalamic seeding to investigate potential treatment targets for fatigue in the present study.

In this study, we hypothesized that (1) alterations in thalamic‐surface FC are associated with fatigue symptoms in CFS, and (2) FC‐guided analyses could identify surface regions that may serve as candidate targets for NIBS. Therefore, we recruited 100 CFS patients and 100 healthy controls (HC) and performed bilateral thalamic seeding to identify candidate surface targets based on FC values. Two approaches were employed: (1) normative analysis, which identified positive and negative peaks using a public dataset of 1000 healthy individuals; and (2) individual analysis, which identified peaks based on subject‐specific features. The identified peaks were then examined in relation to fatigue severity, measured by the Fatigue Scale 14‐items questionnaire (FS14), to determine potential surface targets. In addition, site stimulation simulations were conducted using finite element analysis (FEA) to estimate surface areas that could be influenced by NIBS. Finally, using an additional longitudinal dataset of 35 CFS patients undergoing one month of Tai Chi exercise, we examined whether exercise‐induced changes in FC within the influenced areas were associated with corresponding changes in FS14 scores. Taken together, this study aims to explore the potential of FC‐guided targeting based on thalamic seeding in CFS and to identify candidate cortical targets, which may inform future NIBS strategies for the treatment of fatigue symptoms.

## 2 Materials and Methods

### 2.1 Participants

A total of 100 CFS patients and 100 HC were recruited through public advertisements at Dongzhimen Hospital, Beijing University of Chinese Medicine, and Beijing Anding Hospital, Capital Medical University, between January 2020 and July 2025. Demographic information is summarized in Table 1. The study was approved by the ethics committees of Dongzhimen Hospital (approval number DZMEC‐KY‐2019‐195) and Beijing Anding Hospital (approval number 2024184‐FS‐89), in accordance with the Declaration of Helsinki (2013). Written informed consent was obtained from all participants. The trial was registered in the Chinese Clinical Trail Registry (ChiCTR2000032577 and ChiCTR2200063077).

**TABLE 1:** Demographics of included participants.

	CFS patients	HCs	𝒕∕𝝌 value	*p* value
*N*	100	100	/	/
Age	33.22 ± 9.87	31.24 ± 10.59	1.37	0.173
Gender (male/female)	28 / 72	27 / 73	0.11	0.74
BMI	22.79 ± 2.80	21.46 ± 3.07	1.76	0.079
HAMD	5.81 ± 4.97	4.96 ± 3.54	1.44	0.152
FS14	10.35 ± 2.90	4.73 ± 3.96	10.68	<0.001*

*Note*: The symbol * represents a significant result with *p* < 0.05.

Abbreviations: BMI, body mass index; HAMD, Hamilton rating scale for depression; FS14, fatigue scale 14 items.

The diagnosis of CFS was based on the most widely used definition of 1994 Fukuda CDC criteria (Fukuda et al. 1994). Participants were classified as having CFS if they met the following conditions: (1) clinically unexplained, persistent fatigue lasting for more than six months, which was not the result of ongoing exertion and was not substantially alleviated by rest; and (2) the presence of four or more of the following concurrent symptoms: significant memory impairment or difficulty concentrating, sore throat, tender cervical or axillary lymph nodes, muscle pain, multi‐joint pain without redness or swelling, severe headache, unrefreshing sleep, or post‐exertional fatigue lasting more than 24 h.

CFS patients were eligible if they were 25–65 years old, right‐handed, had not taken psychotropic medications within the past month, and had no contraindications to MRI. HC met the same criteria but did not have CFS. Participants were excluded if they met any of the following conditions: (1) primary diseases that could cause secondary fatigue, such as hypothyroidism or hepatitis B/C infection; (2) fatigue attributable to medication side effects; (3) a history of emotional or psychiatric disorders, such as delusional disorder or anorexia nervosa; (4) major diseases affecting the heart, kidney, lung, liver, or brain, or severe hepatic or renal dysfunction; (5) severe obesity, defined as a body mass index (BMI, calculated as weight [kg] / height^2^ [m^2^]) > 45; (6) pregnancy, breastfeeding, or menstruation; or (7) significant cranial anatomical asymmetry or definitive lesions detected on MRI.

### 2.2 Clinical Measurements

Fatigue severity was assessed using the 14‐item Fatigue Scale (FS14), a widely used instrument for assessing fatigue severity in clinical and research settings originally developed by Chalder et al. (1993). The scale consists of two factors, including physical fatigue (8 items) and mental fatigue (6 items), and has demonstrated good psychometric properties with satisfactory internal consistency and construct validity. Higher scores indicate greater fatigue severity. Depressive symptoms were assessed using the 24‐item Hamilton Rating Scale for Depression (HAMD), in which each item is scored from 0 to 4, and a total score above 20 suggests potential depression. HAMD scores were applied to exclude participants with possible depression. As shown in Table 1, none of the participants in this study exhibited notable depressive symptoms or tendencies.

### 2.3 MRI Acquisitions

Images were acquired using a 3‐Tesla Siemens PRISMA scanner (Siemens Medical Systems, Erlangen, Germany) at Beijing Anding Hospital and a 3‐Tesla Siemens MRI scanner (Germany) at Dongzhimen Hospital. Parameters of the T1‐weighted structural imaging sequence were as follows: repetition time = 1900 ms, echo time = 2.53 ms, field of view = 250 × 250 mm^2^, matrix = 256 × 256, slice thickness = 1 mm. Parameters of the resting‐state echo‐planar imaging sequence were as follows: repetition time = 2000 ms, echo time = 30 ms, flip angle = 90^°^, field of view = 240 × 240 mm^2^, matrix = 64 × 64, slice thickness = 3.5 mm, volumes = 240. The same MRI sequences were used at both hospitals to allow for data combination analysis.

### 2.4 Data Preprocessing

Images of each subject were preprocessed through the frameworks of fMRIPrep 24.1.1 (Esteban et al. 2019) and XCP‐D 0.10.0 (Mehta et al. 2024). Specifically, T1‐weighted structural images were first corrected for intensity non‐uniformity and were then skull‐stripped and segmented into cerebrospinal fluid, white matter, and gray matter. Brain surface reconstruction was performed to enhance the detailed boundary segmentation of these three brain tissues. Next, a volume‐based spatial normalization using nonlinear registration was applied to transform the T1 images from native space to the standard Montreal Neurological Institute (MNI) space. For functional images, removal of the first 10 time points and slice timing correction were performed, and a reference volume was generated. The reference volume went through the steps of head motion correction and co‐registration with the native T1 image, and all functional volumes followed the same steps. Then, several confounding time‐series were calculated, including head motion displacement, signals of cerebrospinal fluid, white matter, gray matter, whole brain, and other component signals. Further, all functional volumes were registered and resampled to the standard MNI 2 mm space.

Subsequently, processed functional volumes with head motion displacement greater than 0.5 mm were censored and then regressed out 36 nuisance regressors, including six motion parameters, mean global signal, mean white matter signal, mean cerebrospinal fluid signal with their temporal derivatives, and quadratic expansion of six motion parameters, tissue signals, and their temporal derivatives, in order to facilitate the reliability of functional signals (Ciric et al. 2017). Finally, the band‐pass filtering between 0.01 and 0.1 Hz and the smoothing with a Gaussian kernel of 6 mm was performed, and the denoised time‐series functional volumes in the MNI space were prepared.

### 2.5 Normative and Individual Analyses

The bilateral thalamus from the Brainnetome atlas (Fan et al. 2016), with label indices from 231 to 246, was used as a combined seed for FC map computation. For each subject, the Pearson's correlation coefficient was calculated between each voxel in the denoised time‐series functional volumes and the averaged signal of the bilateral thalamus. These correlation values were then transformed to *z* values using the Fisher *r*‐to‐*z* transformation, resulting in an FC map with two peaks (one positive and one negative) for each subject.

**FIGURE 1:**
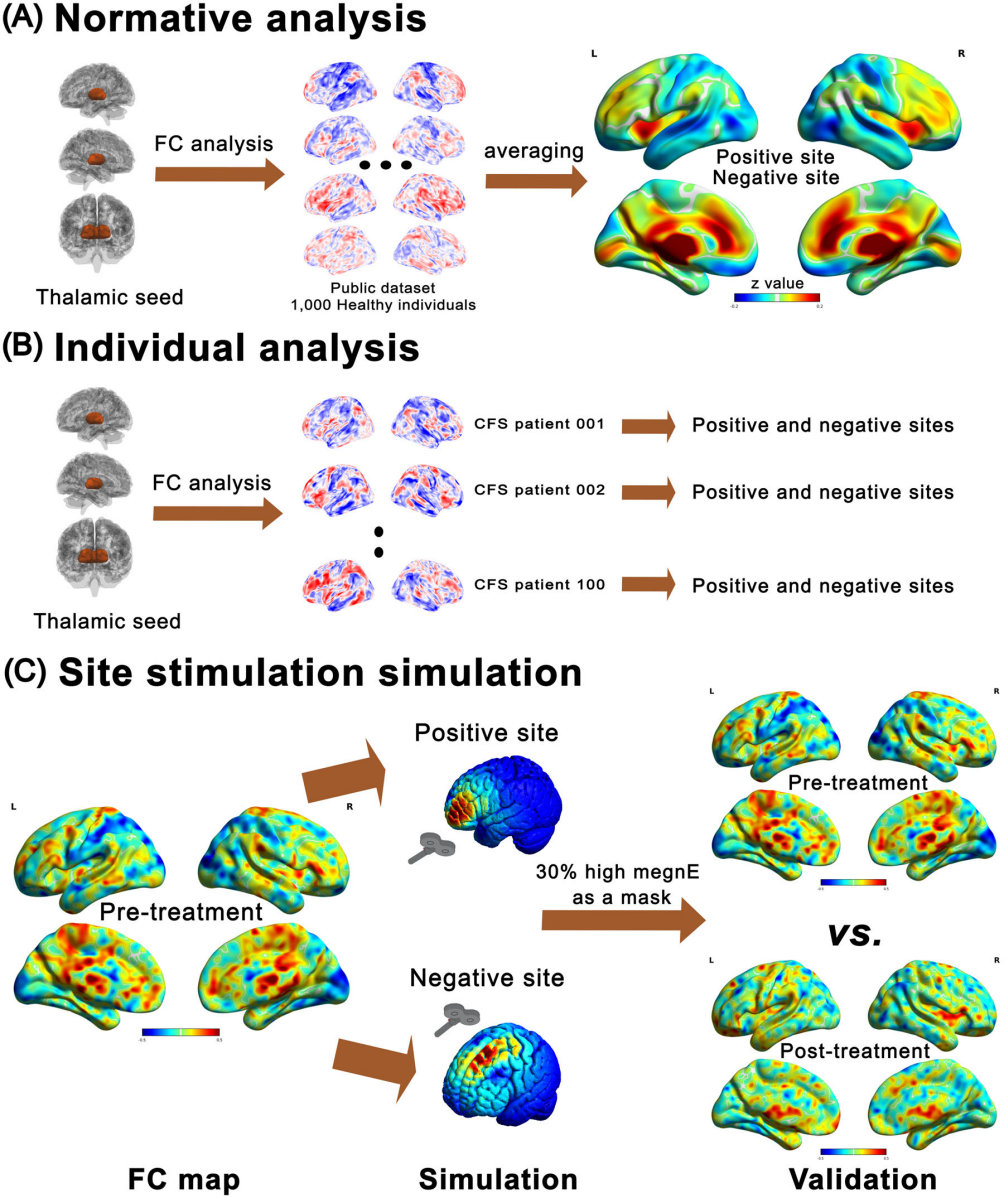
Flowchart of the study. (A) Normative analysis: Thalamic seeding was used to create FC maps based on a large population, which were then averaged to generate a normative FC map and identify positive and negative sites at the normative level. (B) Individual analysis: Thalamic seeding was used to generate FC maps for each CFS patient, from which positive and negative sites were identified at the individual level. (C) Site stimulation simulation: Virtual stimulation was performed on CFS patients prior to treatment by targeting the positive and negative sites identified in both normative and individual analyses. Cortical regions receiving the top 30% of megnE values were extracted as a surface mask, and mean FC values within this mask were compared before and after the intervention to validate the optimal stimulation sites.

The public dataset of 1000 subjects from the Brain Genomics Superstruct Project (GSP) was used (Holmes et al. 2015), which is a widely used large‐scale dataset of healthy individuals. These healthy individuals were aged 18 to 36, and each had one or two preprocessed resting‐state denoised time‐series functional volumes. Briefly, the preprocessed steps included discarding the first four time points, slice timing correction, motion correction, normalization, filtering, and global signal regression. All functional volumes of each subject were combined to compute an FC map, and the 1000 individual FC maps were averaged to generate a normative FC map, as shown in Figure 1A. Nilearn 0.11.1 was used to transform the normative FC map from volume space to fsaverage5 space, and the positive and negative peaks in surface space were identified. Subsequently, the FC values at the identified peaks for the 100 CFS patients were extracted to investigate their relationship with fatigue severity. To enhance the stability of peak comparisons, each peak vertex was expanded to a surface‐based sphere of 6 mm. Finally, both vertex level and sphere level results were presented.

The steps of the individual analysis were the same as those of the normative analysis but were performed on the 100 CFS patients recruited in this study, as shown in Figure 1B. Positive and negative peaks were identified within the cortical surface without any restrictions. Similarly, both vertex level and sphere level results were reported to illustrate individual variability.

### 2.6 FEA‐Based Simulation of Potential Stimulation Effects

Currently in this study, no CFS patients have received NIBS treatment to validate the optimal targeting sites identified through normative and individual analysis. To provide preliminary support for the potential relevance of these sites, we assessed FC alterations in 35 CFS patients recruited from Dongzhimen Hospital who had completed a one‐month Tai Chi exercise program. The protocol and efficacy of this exercise intervention are detailed in our previous work (Wu et al. 2022).

Site stimulation simulations based on FEA were performed using SimNIBS 4.1.0 on the Python 2.8.2 platform. The steps for FEA‐based simulation were as follows: (1) T1 and T2 images of the MNI template were segmented into cortex and scalp, and both cortical and scalp mesh models were reconstructed. The MagVenture MRI B91 coil was used for E‐field simulation, and a direction reference coordinate (MNI [0, –14, 18]) located in the brain center was used to ensure that stimulation was perpendicular to the scalp. (2) Normative and individual FC peaks were resampled onto the scalp using the nearest neighbor method, serving as the stimulation sites for the virtual TMS coil. (3) Virtual TMS stimulations were performed on the 35 CFS patients in the pre‐treatment. (4) Cortical regions receiving the top 30% of electric field magnitude (megnE, units: V/m) were extracted to create a cortical mask. (5) Mean FC values within the cortical mask of the 35 CFS patients were compared before and after the exercise intervention. The flowchart of this procedure is shown in Figure 1C.

### 2.7 Statistical Analysis

All statistical analyses were conducted using Python 2.8.2 and R 4.5.1. Independent *t*‐tests were used to compare CFS patients and HC. Pearson's correlation analyses were performed to examine the relationships between FC and fatigue severity in CFS patients, as well as the relationships between changes in FC and changes in fatigue severity following intervention. All tests were two‐tailed, with a significance level set at 0.05.

## 3 Results

### 3.1 Results of Normative Analysis

The MNI coordinate of the negative site from the normative analysis was (MNI: –51.0, –77.5, 7.0) in the cortex and (MNI: –65.1, –88.4, 7.8) on the scalp, corresponding to the left lateral occipital cortex (label 201 in the Brainnetome atlas), and located near the PO7 electrode according to the 10/10 electroencephalogram (EEG) system defined by Jurcak (Jurcak et al. 2007). The MNI coordinate of the positive site was (MNI: 2.8, 29.7, 26.5) in the cortex, (MNI: 8.5, 69.8, 53.2) on the scalp, corresponding to the right cingulate gyrus (label 180 in the Brainnetome atlas), and located near the Fz electrode.

**FIGURE 2:**
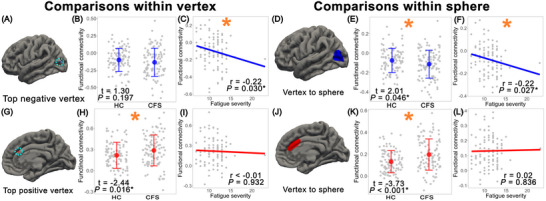
Results of normative analysis. (A) View of the negative vertex. (B) Group comparison of FC values between recruited CFS patients and HC. (C) Correlation between FC and fatigue severity in CFS patients. (D) View of the negative sphere. (E) Group comparison of the negative sphere. (F) Correlation test of the negative sphere. (G) View of the positive vertex. (H) Group comparison of FC values between recruited CFS patients and HC. (I) Correlation between FC and fatigue severity in CFS patients. (J) View of the positive sphere. (K) Group comparison of the positive sphere. (L) Correlation of the positive sphere.

As shown in Figure 2, for the negative vertex, no significant group difference was observed in FC values between CFS patients and HC (*t* = 1.30, *p* = 0.197), while FC values were anticorrelated with fatigue severity in CFS patients (*r* = –0.22, *p* = 0.030). When the vertex was expanded to a 6 mm surface sphere, both the group difference (*t* = 2.01, *p* = 0.046) and the anticorrelation (*r* = –0.22, *p* = 0.027) reached significance. For the positive vertex, a significant group difference in the FC values was found between CFS patients and HC (*t* = –2.44, *p* = 0.016), whereas FC values were not significant associated with fatigue severity in CFS patients (*r* < 0.01, *p* = 0.932). Expanding this vertex to a 6 mm sphere, the group difference (*t* = –3.73, *p* < 0.001) and the correlation (*r* = 0.02, *p* = 0.836) remained unchanged.

These results suggest that the negative site in the left lateral occipital cortex may be more closely related to fatigue severity in CFS, whereas the positive site primarily reflects group differences between patients and controls.

### 3.2 Simulation Results of Normative Sites

**FIGURE 3:**
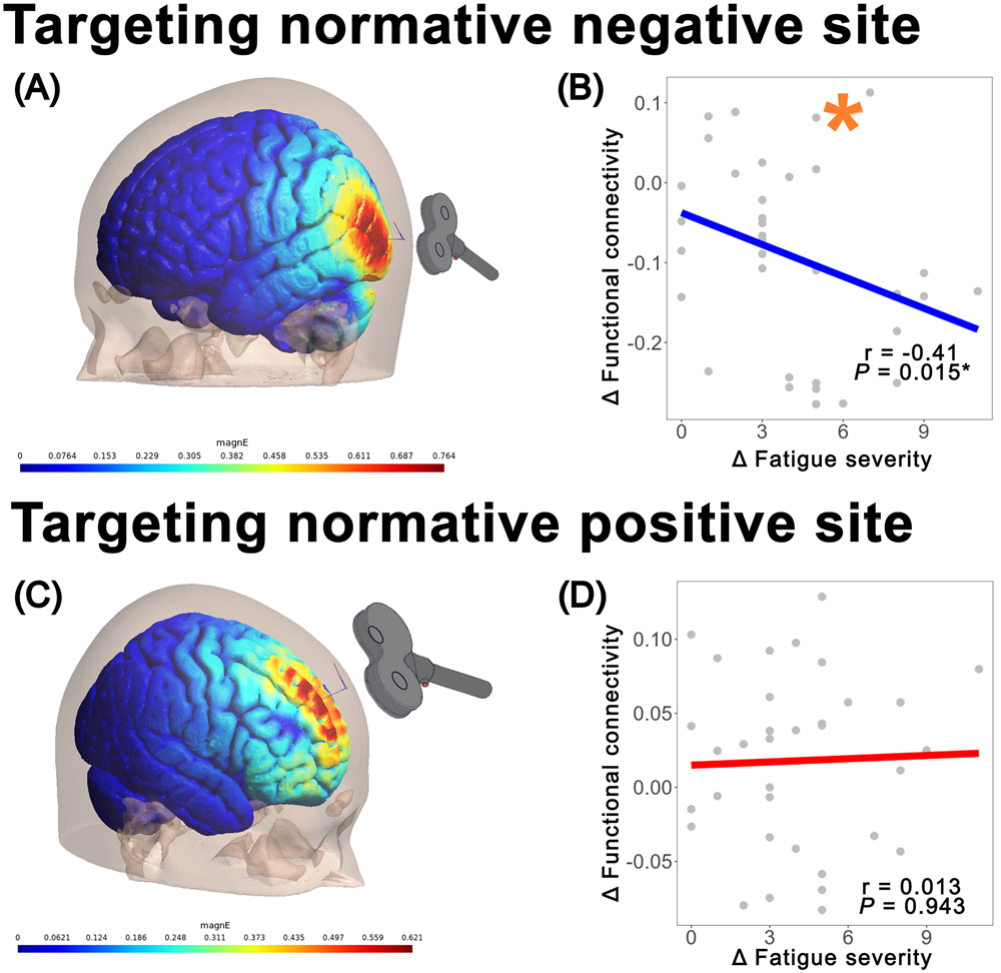
Simulation results of normative sites. (A) View of site stimulation simulation targeting the negative site. (B) Correlation between changes in FC values and changes in fatigue severity in CFS patients after treatment. (C) View of site stimulation simulation targeting the positive site. (D) Correlation between changes in FC values and changes in fatigue severity in CFS patients after treatment.

As shown in Figure 3, when targeting the negative site and selecting the cortical region receiving the top 30% of magnE as the mask, changes in FC values following exercise treatment were anticorrelated with changes in fatigue severity (*r* = –0.41, *p* = 0.015). In contrast, no significant relationship was observed when targeting the positive site (*r* = 0.013, *p* = 0.943). These findings further suggest that the negative site may be more closely related to fatigue‐related changes in CFS than the positive site.

### 3.3 Results of Individual Analysis

**FIGURE 4:**
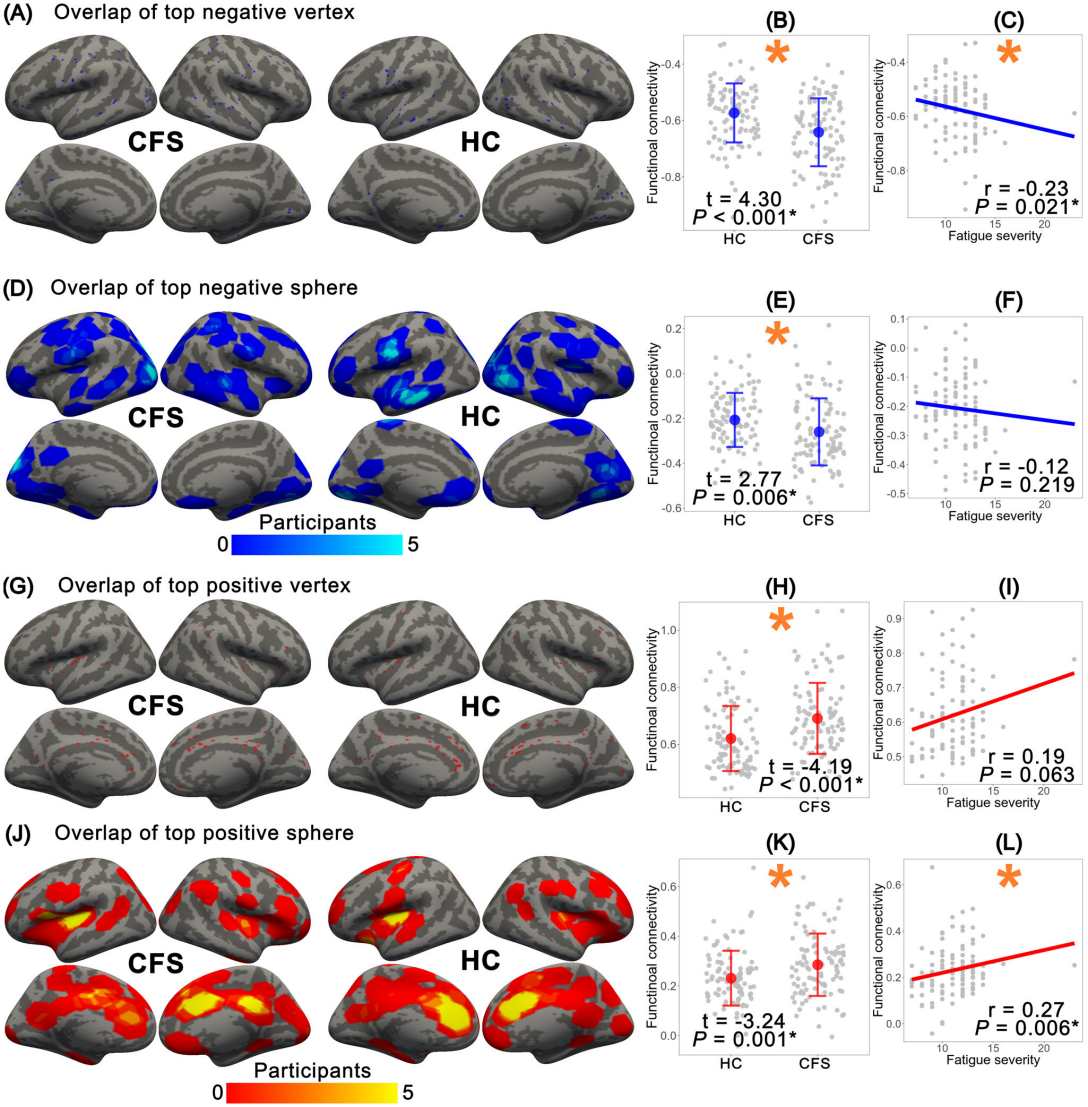
Results of individual analysis. (A) Overlap view of the negative vertex in CFS and HC, respectively. (B) Group comparison of FC values between CFS and HC. (C) Correlation between FC values and fatigue severity in CFS patients. (D) Overlap view of the negative sphere. (E) Group comparison of negative sphere values. (F) Correlation between negative sphere values and fatigue severity in CFS patients. (G) Overlap view of the positive vertex. (H) Group comparison of positive vertex values. (I) Correlation between positive vertex values and fatigue severity in CFS patients. (J) Overlap view of the positive sphere. (K) Group comparison of positive sphere values. (L) Correlation between positive sphere values and fatigue severity in CFS patients.

As shown in Figure 4, because no restrictions were applied in selecting FC peaks, the negative and positive vertices were distributed across the entire brain, reflecting substantial individual variability. Even after expanding each vertex to a sphere, this variability remained pronounced, with an overlap rate of less than 8% among the 100 participants in each group.

Despite the individual variability, the negative vertex showed a significant group difference in FC values between CFS patients and HC (*t* = 4.30, *p* < 0.001), and FC values were anticorrelated with fatigue severity in CFS patients (*r* = –0.23, *p* = 0.021). However, when the vertex was expanded to a sphere, the group difference remained significant (*t* = 2.77, *p* = 0.006), but the anticorrelation was no longer observed (*r* = –0.12, *p* = 0.219). For the positive vertex, a significant group difference in FC values was also observed between CFS patients and HC (*t* = –4.19, *p* < 0.001), but FC values were not correlated with fatigue severity (*r* = 0.19, *p* = 0.063). After expanding the vertex to a sphere, both the group difference (*t* = –3.24, *p* = 0.001) and the correlation with fatigue severity (*r* = 0.27, *p* = 0.006) became significant.

These findings suggest that individual analyses are strongly influenced by inter‐individual variability, leading to heterogeneous peak locations and variable associations between FC values and fatigue severity.

### 3.4 Simulation Results of Individual Sites

**FIGURE 5:**
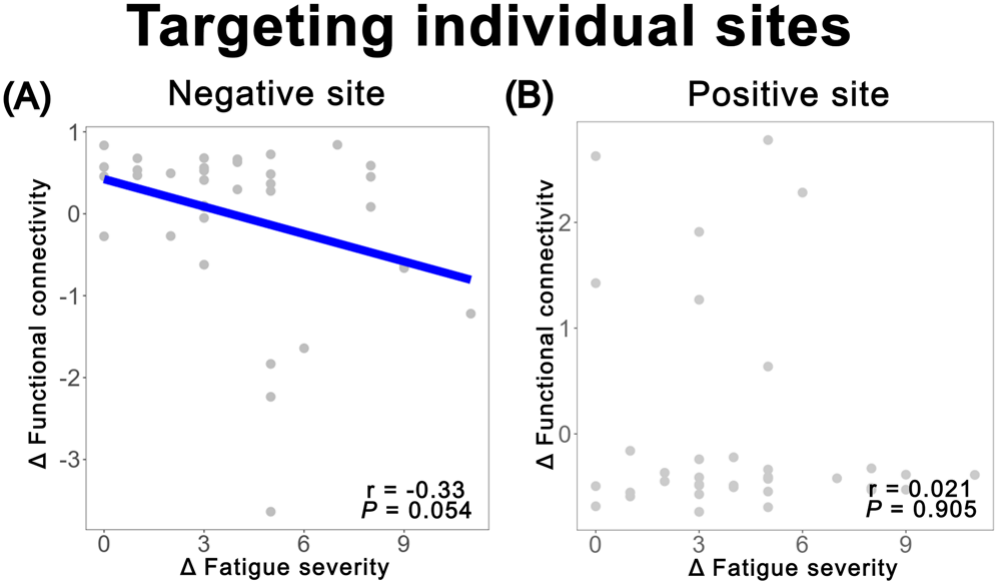
Simulation results of individual sites. (A) Correlation between changes in FC values and changes in fatigue severity in CFS patients after treatment when targeting the negative site. (B) Correlation between changes in FC values and changes in fatigue severity in CFS patients after treatment when targeting the positive site.

As shown in **Figure** **5**, no significant relationship was observed when targeting either the negative site (*r* = –0.33, *p* = 0.054) or the positive site (*r* = 0.021, *p* = 0.905). Together with the marked spatial variability observed in the individual analysis, these results suggest that individually derived stimulation sites may lack sufficient consistency to reliably capture symptom‐related FC changes. In contrast, normative analysis may offer a more spatially consistent framework for target identification.

## 4 Discussion

In this study, we applied both normative and individual analyses in 100 CFS patients and 100 HC to examine whether alterations in thalamic–surface FC are associated with fatigue symptoms in CFS and whether FC‐guided analyses could identify cortical regions that may serve as candidate targets for NIBS. To enhance the robustness of the findings, FC peaks were evaluated at both the vertex and sphere levels. The normative analysis identified a negative peak at the coordinate (MNI: –51.0, –77.5, 7.0) in the left lateral occipital cortex, near the PO7 electrode, whose FC values were significantly associated with both fatigue severity and exercise‐induced changes in fatigue, whereas the positive peak primarily reflected group differences between CFS patients and HC. In contrast, the individual analysis revealed substantial spatial variability in both negative and positive peaks across participants, with very low overlap even after spatial expansion, and the associations with fatigue severity and group differences were inconsistent. These findings suggest that, in the absence of reliable approaches for individualized target identification, our normative analysis may serve as a practical reference framework for guiding target selection and hypothesis generation in future NIBS studies of CFS.

The lateral occipital cortex has been increasingly implicated in fatigue‐related neural processes across multiple neurological conditions, although it is likely involved as part of a broader distributed network rather than serving as an isolated fatigue‐specific region. Functionally, the occipital cortex interacts with frontal regions within attention‐related networks that support both bottom‐up and top‐down attentional processes (Katsuki and Constantinidis 2014). Disruptions within this network may impair attentional control and increase cognitive effort, thereby contributing to the development of mental fatigue. Accumulating evidence supports an association between abnormalities in the occipital cortex and fatigue symptoms across various conditions. For instance, in patients with Parkinson's disease, cerebral blood flow in the occipital cortex increases in parallel with fatigue severity, and the magnitude of this increase correlates with subjective fatigue levels (Liu et al. 2022). Post‐stroke patients with fatigue show reduced resting‐state activity in the occipital cortex compared to those without fatigue (Cotter et al. 2021). Similarly, in patients with ankylosing spondylitis, fatigue severity is associated with gray matter volume in the occipital cortex, while the integrity of the occipital fasciculi is disrupted in those with severe fatigue (Wu et al. 2014). Based on such findings, several studies have begun exploring cortical targets for fatigue‐related neuromodulation. A randomized double‐blind tDCS trial in multiple sclerosis demonstrated that stimulating the parietal cortex improved attention but failed to alleviate fatigue (Hanken et al. 2016). In contrast, another study found that targeting the primary somatosensory cortex with an occipital cathode significantly improved fatigue symptoms in the same patient group (Cancelli et al. 2018). Additionally, tDCS targeting the DLPFC alleviated both fatigue and pain symptoms in ME/CFS patients, whereas stimulation near the occipital nerve (C2 electrode) improved only pain (To et al. 2017). Among various NIBS techniques, tDCS has shown promising therapeutic potential for fatigue. However, the optimal cortical target for achieving consistent clinical benefit remains unclear.

In the present study, using thalamic seeding, we identified a stimulation site located in the left lateral occipital cortex (MNI coordinate [–51.0, –77.5, 7.0]), near the PO7 electrode. FC at this site showed significant associations with both fatigue severity (*r* = –0.22, 95% CI [–0.39, –0.02], *p* = 0.027) and exercise‐induced changes in fatigue following Tai Chi practice (*r* = –0.41, 95% CI [–0.59, –0.14], *p* = 0.015). These correlations correspond to small‐to‐moderate effect sizes, suggesting that this region may contribute to fatigue‐related neural processes within a broader thalamocortical network. Importantly, this site was identified through a data‐driven normative connectivity approach rather than predefined anatomical assumptions. Although these associations reached statistical significance, the observed effect sizes suggest that the identified region likely represents one component of a distributed fatigue‐related network rather than a unique determinant of fatigue in CFS. Therefore, the present findings should be interpreted as identifying candidate cortical targets for future NIBS studies. Taken together, these results suggest that the left lateral occipital cortex may represent a promising candidate region for further investigation in fatigue‐targeted neuromodulation studies. By integrating connectivity‐based targeting with symptom‐related associations, this work provides a methodological framework that may help guide future NIBS research aimed at alleviating fatigue symptoms in CFS and related disorders.

The normative and individual analyses in our study were inspired by research applying TMS in major depressive disorder (MDD). These studies established a research paradigm for NIBS, proposing that stimulation at specific cortical sites can modulate brain connectivity, influence disease‐relevant downstream circuits, and thereby improve clinical symptoms (Cash and Zalesky 2024). Studies have shown that stimulation at the F3 electrode, corresponding to the left DLPFC, serves as a reliable, one‐site‐fits‐all TMS target for MDD across individuals (Siddiqi et al. 2022). Moreover, clinical improvements following TMS at F3 have been linked to changes in FC between the stimulation site and the subgenual cingulate cortex (Zalesky et al. 2019). At the same time, increasing evidence suggests that FC reflects an individual's unique neural architecture. Accordingly, individualized targeting strategies have been proposed as a promising approach for precision neuromodulation and have shown improved efficacy compared with the standard F3‐based protocol in some studies (Cash et al. 2021). However, the practical implementation of individualized targeting remains challenging, as single‐subject connectivity estimates can be influenced by inter‐individual variability and the relatively limited reliability of clinical MRI data.

Inspired by these findings, we compared stimulation sites derived from normative and individual analyses. To address the relatively low signal‐to‐noise ratio inherent in clinical MRI sequences, stimulation sites were visualized at both the vertex and sphere levels to enhance interpretability. Our results showed that the negative and positive peaks identified in the individual analysis exhibited significant group differences between CFS patients and HC, suggesting potential associations between these peaks and fatigue‐related processes. Nevertheless, the relationships between these peaks and fatigue severity were variable, and no significant correlations were observed between changes in these peaks and improvements in fatigue. These findings indicate that individually derived targets may capture meaningful disease‐related signals but may also be strongly influenced by individual variability. Therefore, while individualized targeting remains an attractive long‐term goal for precision neuromodulation, more robust analytical approaches and higher‐quality data may be required before it can be reliably implemented in clinical practice. In this context, normative analysis may provide a practical reference framework for identifying candidate stimulation sites. Specifically, the PO7 electrode, corresponding to the left lateral occipital cortex, represents an easily localized and practically applicable site that could serve as a starting point for future research on NIBS‐based interventions for fatigue symptoms.

Several limitations of this study should be acknowledged. First, the causal validation of our findings was based on exercise treatment rather than direct NIBS intervention, which may limit the reliability of the stimulation site identified in our analysis. Second, the cross‐sectional patients were recruited from two hospitals, whereas the longitudinal cohort was drawn from a single hospital, potentially introducing center‐related bias in our results. Third, the individual analysis employed a relatively simple method to localize potential treatment targets, which may have obscured the true effects of personalized stimulation site selection. Fourth, the use of the older and broader 1994 Fukuda criteria may introduce heterogeneity and limit the generalizability of our findings, and further validation in CFS patients defined by more recent diagnostic criteria is warranted. Fifth, the age ranges of the young healthy individuals in the GSP dataset (18–36 years) and the CFS patients in our study (25–65 years) are not matched, which may reduce the accuracy of stimulation site identification based on the normative analysis. Further validation of the identified normative target is warranted. Despite these limitations, this study highlights the potential of using NIBS to alleviate fatigue symptoms and provides valuable groundwork for future clinical and methodological advancements in this field.

## 5 Conclusion

In conclusion, using bilateral thalamic seeding, we identified a candidate cortical site in the left lateral occipital cortex and near the PO7 electrode, whose FC was associated with both fatigue severity and exercise‐induced fatigue changes in CFS patients. These findings provide new insight into thalamocortical connectivity alterations related to fatigue in CFS. Importantly, this FC‐guided approach may offer a practical starting framework for future NIBS studies aiming to refine stimulation targeting for fatigue symptoms.

## Author Contributions

Kang Wu: conceptualization, methodology, software, data curation, formal analysis, validation, visualization, writing – original draft, writing – review and editing, investigation. Sitong Feng: writing – original draft, writing – review and editing, visualization, validation, data curation. Hongxiao Jia: conceptualization, investigation, funding acquisition, writing – original draft, writing – review and editing, validation, project administration, supervision. Zhuoran Li: writing – original draft, writing – review and editing, visualization, validation. Tian Zhou: writing – original draft, writing – review and editing, data curation, validation. Ziyao Wu: writing – original draft, writing – review and editing, data curation, visualization, validation. Yanzhe Ning: writing – original draft, writing – review and editing, visualization, validation. Kuangshi Li: conceptualization, investigation, funding acquisition, writing – original draft, writing – review and editing, project administration, supervision, validation.

## Funding

This research was supported by the National Natural Science Foundation of China from Beijing Anding Hospital (Grant No.82174311) and the National Natural Science Foundation of China from Dongzhimen Hospital (Grant No.82004437).

## Conflicts of Interest

The authors declare no competing financial interests.

## Data Availability

Data will be made available on request.
